# Exploring the Therapeutic Potential of Royal Jelly in Metabolic Disorders and Gastrointestinal Diseases

**DOI:** 10.3390/nu16030393

**Published:** 2024-01-29

**Authors:** Hesham R. El-Seedi, Suzy Salama, Aida A. Abd El-Wahed, Zhiming Guo, Alessandro Di Minno, Maria Daglia, Chuan Li, Xiao Guan, Daniele Giuseppe Buccato, Shaden A. M. Khalifa, Kai Wang

**Affiliations:** 1 Pharmacognosy Group, Department of Pharmaceutical Biosciences, Uppsala University, Biomedical Centre, P.O. Box 591, SE-751 24 Uppsala, Sweden; 2 School of Food and Biological Engineering, Jiangsu University, Zhenjiang 212013, China; guozhiming@ujs.edu.cn; 3 International Joint Research Laboratory of Intelligent Agriculture and Agri-Products Processing, Jiangsu University, Zhenjiang 210024, China; 4 Indigenous Knowledge and Heritage Center, Ghibaish College of Science and Technology, Ghibaish 51111, Sudan; s.salama999@hotmail.com; 5 Department of Bee Research, Plant Protection Research Institute, Agricultural Research Centre, Giza 12627, Egypt; aidaabd.elwahed@arc.sci.eg; 6 Department of Pharmacy, University of Napoli Federico II, Via D. Montesano 49, 80131 Naples, Italy; alessandro.diminno@unina.it (A.D.M.); maria.daglia@unipv.it (M.D.); d.buccato@studenti.unina.it (D.G.B.); 7 CEINGE-Biotecnologie Avanzate, Via Gaetano Salvatore 486, 80145 Naples, Italy; 8 School of Food Science and Engineering, Hainan University, Haikou 570228, China; lichuan@hainanu.edu.cn; 9 School of Health Science and Engineering, University of Shanghai for Science and Technology, Shanghai 200093, China; gnxo@163.com; 10 National Grain Industry (Urban Grain and Oil Security) Technology Innovation Center, Shanghai 200093, China; 11 Psychiatry and Neurology Department, Capio Saint Göran’s Hospital, Sankt Göransplan 1, 112 19 Stockholm, Sweden; 12 State Key Laboratory of Resource Insects, Institute of Apicultural Research, Chinese Academy of Agricultural Sciences, Beijing 100093, China

**Keywords:** royal jelly, diabetes mellitus, gastrointestinal diseases, cardiovascular diseases, bioactive compounds

## Abstract

Metabolic disorders, encompassing diabetes mellitus, cardiovascular diseases, gastrointestinal disorders, etc., pose a substantial global health threat, with rising morbidity and mortality rates. Addressing these disorders is crucial, as conventional drugs often come with high costs and adverse effects. This review explores the potential of royal jelly (RJ), a natural bee product rich in bioactive components, as an alternative strategy for managing metabolic diseases. RJ exhibits diverse therapeutic properties, including antimicrobial, estrogen-like, anti-inflammatory, hypotensive, anticancer, and antioxidant effects. This review’s focus is on investigating how RJ and its components impact conditions like diabetes mellitus, cardiovascular disease, and gastrointestinal illnesses. Evidence suggests that RJ serves as a complementary treatment for various health issues, notably demonstrating cholesterol- and glucose-lowering effects in diabetic rats. Specific RJ-derived metabolites, such as 10-hydroxy-2-decenoic acid (10-HDA), also known as the “Queen bee acid,” show promise in reducing insulin resistance and hyperglycemia. Recent research highlights RJ’s role in modulating immune responses, enhancing anti-inflammatory cytokines, and suppressing key inflammatory mediators. Despite these promising findings, further research is needed to comprehensively understand the mechanisms underlying RJ’s therapeutic effects.

## 1. Introduction

Obesity, insulin resistance, hypertension, and heart disease are groups of diseases categorized as metabolic disorders [1]. Metabolic disorders are caused by the disruption of regular metabolic functions triggered by oxidative stress and chronic inflammation. The characteristics of chronic inflammation include elevated levels of inflammatory mediators, such as chemokines and cytokines, which confirm the promotion of the development of metabolic disorders [2]. These chronic inflammations also contribute to the development of major chronic diseases such as non-alcoholic fatty liver disease and obesity. Type 2 diabetes and metabolic disorders now rank among the main risks to human health [3,4]. The significant rise in the prevalence of various metabolic diseases is related to aging, environmental factors, changes in lifestyle, and genetics [5]. Metabolic disorders have been estimated to affect 25% of the population globally [6].

Metformin and Glimepiride are the two synthetic drugs that are currently validated and available for the management of Type 2 diabetes mellitus (T2DM) [7,8]. Metformin is the first-line treatment and has been used for many decades to reduce blood sugar [9]. One of the alternate methods for treating metabolic disorders, and therefore reducing health risks, is utilizing natural product resources. Berberine, derived from the root of *Berberis vulgaris* L. and taken as a typical example, demonstrates significant potential in fighting T2DM [10].

Royal jelly (RJ) is a “rich source of nutrients” that nurse bees produce and feed to worker larvae and queen bees. RJ supplementation is beneficial for a variety of disorders, including diabetes [11,12], gastrointestinal diseases [13,14], and cardiovascular diseases [15,16]. The active ingredients of RJ, including its proteins, carbohydrates, and fats, as well as its minerals, amino acids, vitamins, enzymes, hormones, and polyphenols, are what provide its biological properties [17].

The current review intends to emphasize the protective properties of RJ and/or its components against metabolic disorders such as diabetes, gastrointestinal ailments, and cardiovascular diseases as a part of our ongoing project studying honeybee products [18,19,20].

## 2. Methodology

Sci-finder, PubMed, Google Scholar, Web of Science, ScienceDirect, Microsoft Academic Search, Core, and Scopus were all accessed to conduct a literature search. The following keywords were used to address the search terms: “royal jelly”, “diabetes mellitus”, “gastrointestinal diseases”, and “cardiovascular diseases”. The search included published studies, and therefore only articles in English were selected. Studies that explored the functions of RJ or its active components with the co-administration of prescription products were chosen. The search approach identified 310 specific studies, out of which 87 were disregarded due to the irrelevance of the study scope. Finally, the studies were further analyzed to offer an insightful overview of the field’s progress. The authors, year of publication, RJ utilization, dosage form or percentage, category of investigation, main points, pathway of interaction, and techniques used were all data items.

## 3. Royal Jelly in Diabetes

**Figure 1:**
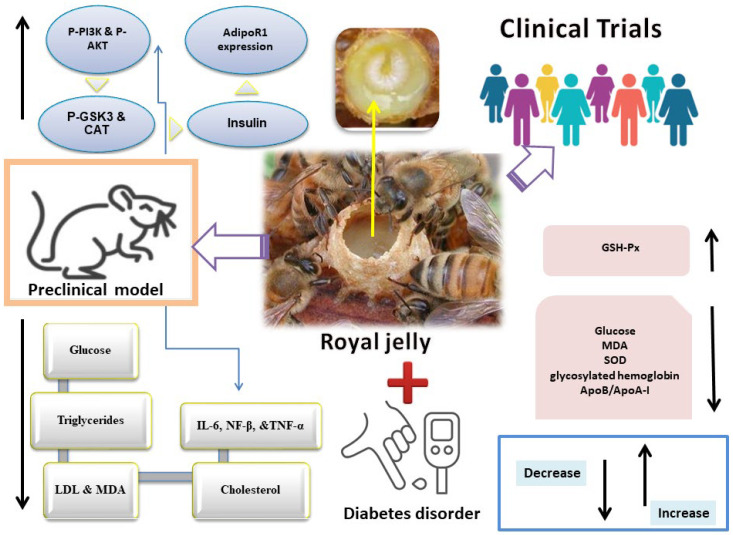
Mechanism of action of royal jelly in diabetes mellitus (DM), preclinical and clinical models [28,29,30,31,32]. LDL: low-density lipoprotein; SOD: superoxide dismutase, IL-6: interlukin-6; MDA: malondialdehyde; GSH-Px: glutathione peroxidase; ApoB/ApoA-I: apolipoprotein B/apolipoprotein A-I; AdipoR1: adiponectin receptor-1; CAT: catalase; p-GSK3β: glycogen synthase kinase 3β; p-AKT: phosphorylated Akt; PI3K: phosphoinositide 3-kinase; IL-1β: interleukin-1β; TNF-α: tumor necrosis factor-α; NF-κB: nuclear factor kappa-B; COX-2: cyclooxygenase-2.

Diabetes mellitus (DM) is a worldwide metabolic disorder. According to Saeedi et al., it affected 463 million individuals globally in 2019, with that figure expected to climb to 578 million by 2030 and 700 million by 2045 [21]. DM is anticipated to become more prevalent in the world, and thus health concerns are only liable to increase [22]. Diabetes is a prevalent health issue that affects both sexes equally and impairs sexual function (e.g., sexual disinclination, negative pregnancy outcomes, infertility, loss of penile erection, and diminished clitoral sensitivity). The male reproductive system is impacted by DM in a variety of anatomical and functional aspects, with reduced sperm parameters being an example of the secondary complications of diabetes [23]. Clinically, T2DM, which accounts for 90% of cases of diabetes, is characterized by hyperglycemia and insulin insufficiency caused by cell dysfunction and insulin resistance (IR) in target organs, including the liver, heart, skeletal muscle, and adipose tissue. Atherosclerosis, coronary heart disease, and kidney disease are all chronic consequences that are highly likely to develop in T2DM patients [24]. The two most common causes of morbidity in people with DM are infections and foot ulcers [25]. Up to 60% of non-traumatic lower-limb amputations in diabetics result from diabetic foot ulcers (DFU), the leading risk factor. Given the high expense of diabetic medical treatments, it is critical to explore alternative entities that can be cost-effective. These choices should not only manage blood glucose levels, but also reduce the probability of complications [26]. Thus, the race to find a sustainable and economically viable solution for diabetes remains ongoing. As demonstrated below, RJ treatments have shown therapeutic potential in both rodent and human diabetic models and are effective against hypercholesterolemia and diabetes, as mentioned in Figure 1 [12,27].

### 3.1. Preclinical Studies

In preclinical studies, RJ was given orally to KK-Ay mice at a dose of 10 mg/kg body weight (BW). In obese/diabetic KK-Ay mice, RJ treatment improves hyperglycemia and partially lowers BW. RJ administration activates the expression of adiponectin (AdipoQ) and adiponectin receptor-1 (AdioR1), which then activate the expression of phosphorylated AMP-activated protein kinase (pAMPK). Additionally, RJ treatment that increases adiponectin receptor-1 (AdipoR1) expression also boosts Ppara and Pgc1a expression, which improves lipid utilization and causes a reduction in BW in KK-Ay mice [28]. Adult male Wistar rats were divided into four groups: diabetic, RJ, diabetic treated with RJ, and control. To induce diabetes, streptozotocin-induced diabetes (STZ)was administered intravenously at a dosage of 60 mg/kgBW. RJ was then administered via gavage at a dosage of 100 mg/kg BW for six weeks. Testicular weight, viability, sperm count, deformity, motility, chromatin quality, DNA integrity, testicular tissue malondialdehyde (MDA) levels, and serum testosterone were all enhanced via RJ in diabetic mice [33]. A total of 28 adult Wistar rats were randomized and divided into four groups: control, RJ, diabetic, and hyperglycemic treated with RJ. To induce diabetes, a single intraperitoneal injection of STZ at a dose of 50 mg/kg BW was used. The rats were then administered RJ (100 mg/kg BW) orally each day for a duration of six weeks. The treatment with RJ resulted in improved levels of catalase (CAT) and ferric reducing antioxidant power (FRAP) when compared to other groups [29]. RJ was given orally to the diabetic rats at a dosage of 100 mg/kg for 42 days following STZ. RJ improved the serum levels of aspartate aminotransferase (AST), alkaline phosphatase (ALP), high-density lipoprotein cholesterol (HDL-c), alanine aminotransferase (ALT), total protein (TP), fasting blood glucose (FBG) levels, insulin, and albumin. RJ dramatically lowered MDA levels in the liver and pancreatic tissues and simultaneously normalized the levels of CAT and FRAP [30]. In a similar experimental study, diabetes was induced in rats through an intraperitoneal injection of STZ (60 mg/kg BW). Then, RJ was administered via gavage for three days at doses of 100 and 200 mg/kg. This treatment notably decreased the levels of cholesterol, glucose, low-density lipoprotein (LDL), and triglycerides in the diabetic rats. Interestingly, the rats that received RJ treatment exhibited significantly elevated HDL levels compared to the untreated diabetic rats [11]. The 18 adult Wistar albino rats were grouped into three groups: control, STZ-induced diabetes, and STZ-induced diabetes plus RJ at a dosage of 400 mg/kg/day for a month. To induce diabetes, STZ (60 mg/kg) was injected intraperitoneally once. Both the RJ-treated and untreated diabetic rats exhibited lower body and testicular weights compared to the control group. Rats treated with STZ had significantly more degenerative alterations in their spermatogenesis and seminiferous tubules, according to the histological analysis. The RJ treatment group, on the other hand, revealed nearly normal morphology, in addition to more intense immunohistochemistry staining for Ki67-positive cells [34]. To induce diabetes, STZ was administered intravenously to rats once at a dosage of 75 mg/kg BW. The rats were categorized into four distinct groups: a healthy control group and three treatment groups. Three groups of rats; untreated diabetic group, 100 mg/kg/daily of metformin group, and one group received a honey–RJ (H–RJ) combination, containing 2% RJ and 98% honey. H–RJ was given daily to this rat group (100 mg/kg BW). The H–RJ treatment significantly lowered the levels of very low-density lipoprotein (VLDL) in the blood, compared to both the control therapy and metformin treatment. Rats with diabetes can effectively present with lower blood sugar when given H–RJ. This combination can also successfully lower triglycerides and VLDL-C lipids (TGs) [35]. Likewise, C57BL/6J mice were subjected to a high-fat diet (HFD) and administered a 5% RJ diet. Alloxan-induced diabetes in male Albino Wistar rats was discussed, as well as the hypoglycemic effect of composite formulations of *Moringa oleifera* seed oil extract and RJ. When compared to pure *M. oleifera* or pure RJ, the medication containing 20% RJ mixed with *M. oleifera* seed extract was found to be more effective in reducing blood sugar levels in treated mice [36]. Taken together, this dietary intervention with RJ effectively mitigated diet-induced obesity, hyperglycemia, and hepatic steatosis in mice by stimulating metabolic thermogenesis in brown adipose tissue (BAT) [37].

**Figure 2:**
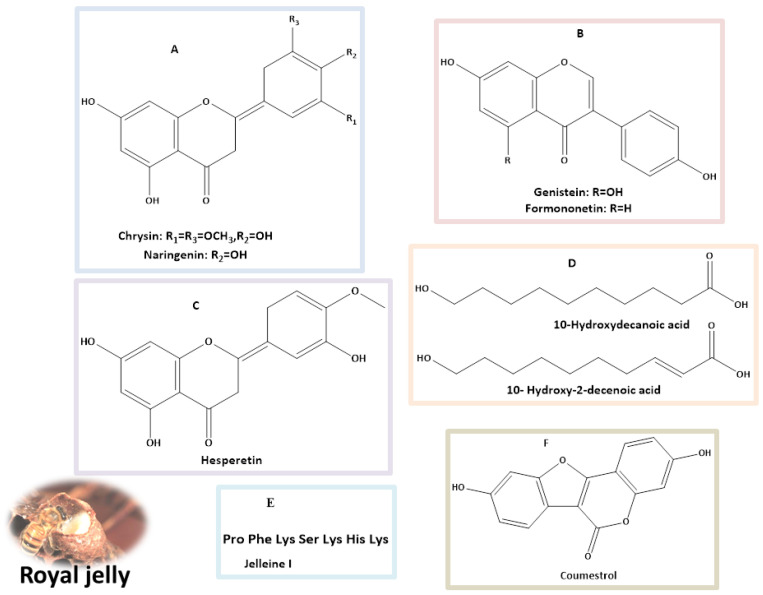
Major classes of natural products (**A**–**C**) flavonoids; (**D**) fatty acids; (**E**) peptide; and (**F**) coumestans identified in royal jelly with potential anti-diabetes properties.

Fatty acid 10-hydroxy-2-decenoic acid (10H2DA) is a component found in RJ (Figure 2, Table 1). Female KK-Ay mice received 10H2DA orally at a dosage of 3 mg/kg BW via gavage for four weeks. It greatly reduced insulin resistance and hyperglycemia. In skeletal muscles, 10H2DA elevated the expression of the pAMPK protein; however, this expression was unrelated to elevated glucose transporter 4 (GLUT4) translocation. Adiponectin receptor mRNA expression was not improved by 10H2DA, and the liver’s glycogen synthetase kinase-3β (GSK-3β) phosphorylation was not triggered by the insulin signaling cascade [38]. The main active component of RJ is 10-hydroxydecanoic acid (10-HDA) (Figure 2). Recent research findings indicate that 10-HDA may possess anti-T2DM properties. When administered orally at a dosage of 100 mg/kg BW, it stopped liver degeneration in the diabetic rats and boosted insulin levels while decreasing fasting blood glucose. Additionally, 10-HDA intervention improved lipid peroxidation, reduced liver NF-β nuclear translocation, reduced interlukin-6 (IL-6) and tumor necrosis factor (TNF-α) content, and elevated P-PI3K, phosphorylated Akt (p-AKT), and glycogen synthase kinase 3β (p-GSK3β( protein levels. It also enhanced glutathione peroxidase (GPx), superoxide dismutase (SOD), and CA activity in diabetic mouse livers. Through the PI3K/AKT/GSK3 signaling pathway, 10-HDA clearly exhibited hypoglycemic effects on diabetic mice [39].Growth factor deficiency and bacterial infection are two of the main factors causing non-healing wounds in diabetics [40]. 8-Bromoadenosine-3′, 5′-cyclic monophosphate (8Br-cAMP) and antimicrobial peptide Jelleine-1 (J-1) (Figure 2) were combined to form a hydrogel without the use of any other gelators or chemical crosslinkers. This hydrogel demonstrated remarkable antibacterial action in a wound model in diabetic rats infected with methicillin-resistant *Staphylococcus aureus* (MRSA) [41].

### 3.2. Clinical Studies

A double-blind, placebo-controlled trial including 50 T2DM patients was carried out, where either 1000 mg of RJ or a placebo were administered to subjects three times per day for eight weeks. The groups were assigned to the RJ or placebo groups at intervals. Baseline characteristics and food intake between groups did not differ significantly. In the RJ group, the mean glucose level decreased (−9.4 mg/dL vs. 4 mg/dL), the mean ApoA-I concentration increased (34.4 mg/dL vs. −1.08 mg/dL), and there was a significant decrease in the mean apolipoprotein B (ApoB) /apolipoprotein A-I (ApoA-I), (0.008 vs. 0.13; *p* < 0.044, respectively) when comparing the RJ group to the placebo group [31].

For eight weeks, 50 female T2DM volunteers were divided into two groups and given either a daily dose of 1000 mg RJ (soft gel) or a placebo. In the RJ group, the average fasting blood sugar dropped significantly to 149.68 mg/dL after supplementation with RJ. The mean serum levels of glycosylated hemoglobin also significantly decreased to 7.05%, and the mean insulin concentration significantly decreased through RJ supplementation to 27.5 pmol/L. MDA levels declined, and GPx and erythrocyte superoxidase dismutase activity was dramatically elevated [32]. A daily dose of a 1000 mg soft gel of RJ or a placebo was administered to 50 female T2DM volunteers, divided into two respective groups, in a randomized clinical trial for 8 weeks. RJ supplementation reduced the daily total energy and carbohydrate intake as well as mean BW (72.45 vs. 71.00 kg) when compared to the control group [12]. Another randomized controlled trial comprised 46 T2DM patients aged 25–65 years with a hemoglobin A1c (HbA1c) of 6–8%. For eight weeks, the patients were randomized to take 1000 mg of RJ supplement or a placebo three times each day. In the RJ group, the insulin resistance index (HOMA-IR) decreased (1.98 vs. 3.13) while the serum total antioxidant capacity increased (907.63 vs. 765.69 mol/L) [57].

The effectiveness of topical RJ for treating diabetic foot ulcers has also been studied [58]. The trial design was randomized, controlled, and open-label, with a 12-week average follow-up time. After conservative debridement of necrotic tissue and irrigation with warm normal saline, 189 eligible patients with diabetic foot wounds from three outpatient clinics in Egypt were randomized to receive a local application of either RJ + Panthenol (PedyPhar^®^ Ointment) or Panthenol ointment underdressing. The purpose of the research was to look at the use of PedyPhar^®^ Ointment in the treatment of individuals suffering from limb-threatening diabetic foot infections [59]. At the end of the 12-week follow-up period, PedyPhar^®^ revealed a greater degree (32.4%) of full healing of limb-threatening wounds in the target population, versus 12% in the Panthenol-treated (control) group [60].

Adiponectin is an adipokine released from adipose tissue that has a role in insulin sensitivity [61], and has been reported for its inhibitory effect on the glucogenesis process in the liver via down-expression of phosphoenolpyruvate carboxykinase and glucose-6-phosphatase genes, which play crucial enzymatic roles in glucose production by the liver [62]. Furthermore, research has shown that giving RJ to diabetic rats at a dosage of 10 mg/kg/day for a month controls hyperglycemia via the acceleration of adiponectin secretion [28]. The possible mechanism of RJ in diabetes was reported by Maleki et al. (2019), through the activation of the AMP-activated protein kinase pathway through increasing the production of adiponectin in skeletal muscle and liver cells [63]. Joshi et al. (2019) noted that the activation of the AMPK protein increases the cells’ uptake of glucose, while reducing intracellular secretion of glucose. Therefore, the AMPK signaling pathway is the target in controlling diabetes [64].

## 4. Royal Jelly in Gastrointestinal Diseases

Gastrointestinal disorders are common illnesses, including irritable bowel syndrome, peptic ulcers, liver diseases, pancreatitis, gallstones, and Crohn’s disease, and are often found in tropical regions [65]. Sperber et al. (2021) estimated that 40% of the people in 33 countries across six continents have functional gastrointestinal problems [66]. A strong link between gastrointestinal diseases and the diet of individuals in at-risk groups has been found. The dietary habits of people in the different areas contribute to the composition of their individual gut microbiota, and this is particularly noticeable when people move from urban to rural areas [67]. According to Rizello et al. (2019), a westernized diet rich in carbohydrates and animal proteins is a main contributor to the development and progression of chronic inflammatory bowel disease [68]. Due to involvement of the gastrointestinal tract in the absorption of nutrients, as well as its role in immune response, the risk of developing an inflammatory, autoimmune, chronic disease is inevitably increasing [69].

RJ is considered one of the most important super foods, having displayed much biological activity in preclinical and clinical studies [70]. As a honey bee product, it has been documented for its active potential against many disorders, including inflammation, liver disease, hypercholesterolemia, oxidative stress, and immune disease [71]. RJ contains many bioactive compounds such as proteins, vitamins, phenolics, and flavonoids. Additional pharmaceutical studies have revealed that the bioactive, major protein constituents of RJ (MRJPs) are considered the main therapeutic compounds of those tested [72,73]. 

### 4.1. Inflammatory Bowel Diseases

Ulcerative colitis and Crohn’s disease are both chronic, inflammatory bowel illnesses. Inflammatory bowel disease describes a persistent, non-infectious inflammation with uncertain causes, affecting one or more locations in the digestive system. According to global estimations, the prevalence of inflammatory bowel disease accounted for approximately 7 million people in 2017 [74]. Hence, the exploration of natural product remedies would pave the way to natural and complementary tools for healing. For instance, the experimental induction of colon inflammation using 2,4,6-trinitrobenzene sulphonic acid was found to be significantly inhibited by the administration of RJ in mice at a dosage of 250 mg/kg/day for a week via the inhibition of pro-inflammatory cytokines, TNF-α, and interlukin-1β (IL-1β) along with the elevation of the anti-inflammatory cytokine interlukin-10 (IL-10) [75]. Another study revealed that daily administration of RJ (150 mg/kg) considerably ameliorated the damage caused by acetic acid in rats with induced colitis, manifesting as decreased lesion areas in the colon where the intestinal mast cells were also involved in inflammation [76]. Likewise, similar doses of RJ were found to reduce the proliferation of T-lymphocytes involved in the intestinal inflammation induced by acetic acid in rats [77]. According to a recent study, synergism between RJ and selenium exhibits significant anti-inflammatory activity in inflammatory bowel disease in mice and promotes intestinal health through the improvement of the gut microbiota [78].

**Figure 3:**
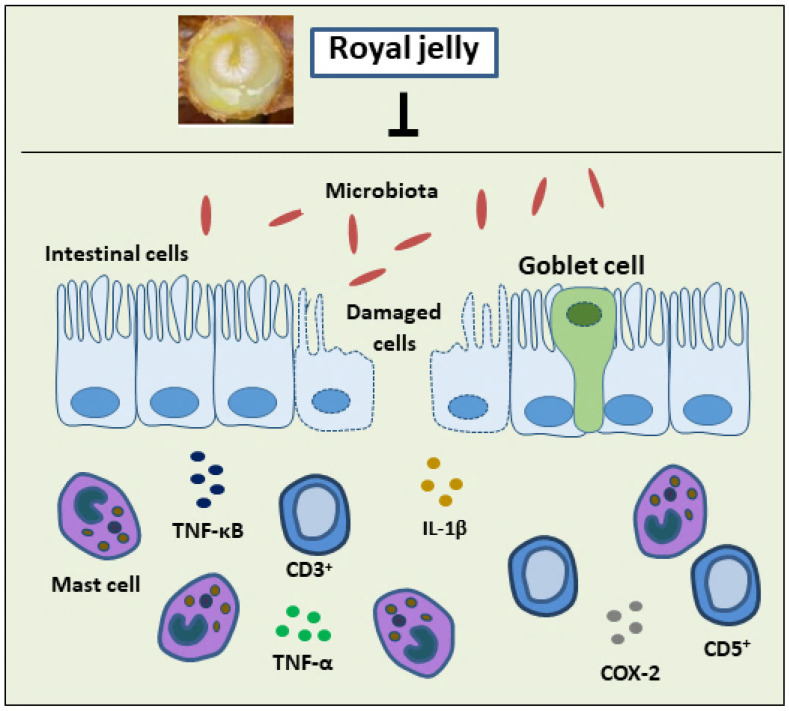
Treatment with royal jelly suppressed the rise of CD3+, CD5+, CD8+ and CD45+ T-cells, pro-inflammatory cytokines, IL-1β, TNF–α, and the expression of major inflammatory mediators (COX-2 and NF-κB) in the colon of rats with colitis. IL-1β: interleukin-1β; TNF-α: tumor necrosis factor-α; NF-κB: nuclear factor kappa-B; COX-2: cyclooxygenase-2.

The mechanism of RJ in treating inflammatory bowel syndrome has been reported by Guo et al. (2022) as shown on Figure 3. The investigation revealed that RJ boosted the activity of the anti-inflammatory cytokine IL-10 and the intracellular antioxidant enzyme GPx. Additionally, RJ decreased the number of CD3+, CD5+, CD8+, and CD45+ T-cells, the release of TNF-αand the pro-inflammatory cytokines IL-1β, the nuclear factor Kappa-B (NF-κB), and cyclogenase-2 (COX-2) and tumor necrosis factor-induced injury in rats with colitis induced by 2,4,6-trinitrobenzene sulfonic acid [79].

### 4.2. Lactose Intolerance

Lactose intolerance is a gastrointestinal disorder that results from a lack of β-galactosidase, resulting in the maldigestion of lactose from milk and milk products. Patients with lactose intolerance present with symptoms such as pain in the abdomen, diarrhea, and flatulence, which appear after the intake of lactose-containing foods [80]. Recently, researchers have found that the synergism between RJ and probiotic yogurt has potent activity in treating lactose intolerance [81].

There is growing evidence that lactose intolerance symptoms can be treated with probiotic bacteria found in fermented and unfermented milk products [82]. The mechanism of RJ in reducing lactose intolerance relies on the activity of probiotics delivered via fermented milk products, which have been found to play an important role in health benefits, as reported by Hassan et al. (2022). The fermentation of milk with 1% RJ displayed the presence of abundant probiotics, namely *Lactobacillus helveticus*, which results in boosting the bioactive properties of fermented milk [83].

### 4.3. Chronic Diarrhea and Constipation

The symptoms of chronic constipation include uncomfortable defecation, marked by straining and difficulty along with extended time in stool passage [84]. Constipation in children is estimated to affect from 1% to 30% of the young generation worldwide [85]. Compared to standard antiviral medication, honey has been shown to reduce the incidence and duration of viral diarrhea [86]. As documented by Miyauchi-Wakuda et al. (2019), under in vitro circumstances, acetylcholine in RJ induced contractions of the smooth muscle of the mouse’s ilium via the muscarinic acetylcholine receptor, which was independent of nicotinic acetylcholine activity. The intake of royal jelly does not result in severe symptoms like diarrhea in normal situations [14,87]. Further, the anti-diarrheal potency of RJ could be attributed to the antimicrobial activity of its peptide constituents, royalisin and royalactin [88,89]. Even though RJ has a high concentration of acetylcholine, only one oral dose of RJ was not enough to boost intestinal motility or alleviate constipation. 

### 4.4. Gastrointestinal Ulcer Disease

Gastric and intestinal ulcers induced by diclofenac (50 mg/kg) have been normalized using RJ at a dose of 150 mg/kg or 300 mg/kg via the increase of prostaglandin-2 (PGE-2) and COX-2 in the stomach tissues of mice, as well as reducing myeloperoxidase (MPO) and inducible nitric oxide synthase (iNOS) [71]. Another study revealed that acetic acid-induced peptic ulcers in rats could be treated significantly using a daily dose of RJ (200 mg/kg), in comparison to the commonly used anti-ulcer drug omeprazole (20 mg/kg), for 14 days of treatment [90]. Furthermore, when RJ was administered to rats at a dose of 250 mg/kg, it protected them from ulcers in the stomach caused by ethanol. This was relative to the ulcer-preventing medication, lansoprazole, given at a dose of 30 mg/kg. The mechanism of gastroprotection has been claimed to be due to the attenuation of pro-inflammatory cytokines, TNF-α, lipid peroxidation, and IL-1β in addition to the augmentation of the endogenous antioxidant enzyme SOD and CAT [13]. El-Naeem and Fareed (2022) reported the positive effects of 30-day administration of RJ (300 mg/kg) on ameliorating the gastric mucosal histopathological changes that were caused in rats by intra-peritoneal injection of 0.5 mg nicotine tartarate [91].

### 4.5. Liver Disease

#### 4.5.1. Preclinical Studies

Synergistic, daily treatment of mice with the drug diclofenac (50 mg/kg) for seven days, following which RJ was given orally at dosages of 150/or 300 mg/kg for a month, was found to alleviate the hepato-renal toxicity of the drug through over-expression of PGE-2 and COX-2 in the animals’ liver and stomach tissues [71]. The hepatic toxicity triggered by the immunosuppressive drug azathioprine was found to be altered by oral administration of RJ (200 mg/kg) in rats through the attenuation of the high levels of serum hepatic enzymes caused by an intra-peritoneal injection of 50 mg/kg dose of azathioprine [92]. According to a recent study, feeding diabetic rats 300 mg/kg of RJ for 16 weeks, development of non-alcoholic fatty liver disease (NAFLD) was reported. The study concluded that RJ has anti-inflammatory and antioxidant properties that protect against NAFLD, while also regulating the metabolism of fatty acids such as arachidonic acid and linoleic acid as well as the production of unsaturated fatty acids [93]. The study revealed that RJ treatment significantly raised the serum levels of adiponectin and concurrently raised the hepatic phosphorylation of 5’ adenosine monophosphate-activated protein kinase (AMPK). Where the hypolipidemic effect of RJ is mediated mainly by regulating AMPK, these effects have been noticed in rats that were fed a high-fat diet and subsequently developed NAFLD. RJ suggests a novel, independent mode of action by promoting fatty acid oxidation via activation of hepatic AMPK signaling and by suppressing cholesterol formation via sterol regulatory element-binding proteins SREBP1/2 without altering the production of adiponectin, the enzyme responsible for fatty acid oxidation, and lowering the synthesis of triglycerides and cholesterol [93,94].

#### 4.5.2. Clinical Studies

**Figure 4:**
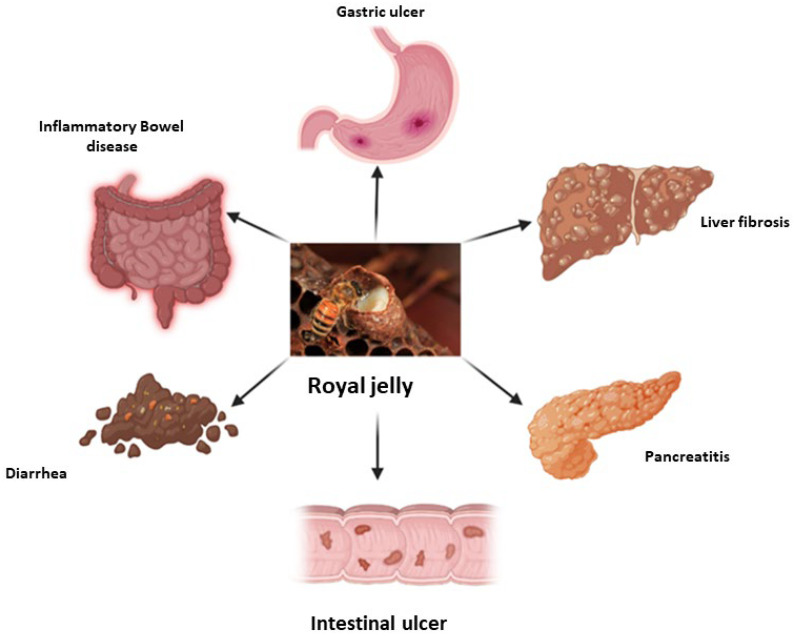
Potential activity of royal jelly in the most common gastrointestinal diseases.

A further investigation found that administration of 1 g/day of RJ for 30 days to patients with chronic hepatitis B could influence the immunological responses of patients by inhibiting the protein responsible for initiating inflammatory responses, NLRP1. Additionally, RJ up-regulated the functions that the inflammasome adaptor speck-like protein (ASC) performs in modulating immune responses [95]. Collectively, RJ was documented for its potent activity in treating gastrointestinal diseases as per in vitro and in vivo studies. The possible impact of RJ on various digestive tract illnesses is summarized in Figure 4.

## 5. Royal Jelly in Cardiovascular Disease

Among all disorders, cardiovascular conditions pose the greatest threat to human health and cause the greatest number of fatalities (more than 17 million/year) [96]. The latest mortality records in Europe have estimated there to be more than 3.5 million deaths per year due to cardiovascular diseases, ranking them first in the world for modern mortality causes [97]. Aslan et al. (2021) have reported that, in the rat model, the administration of RJ to drinking water at doses of 50 or 100 mg/kg for a month demonstrated cardioprotective activity against fluoride-induced heart injury through the down-expression of Bcl-2 protein and the enhanced expression of Bcl-2-associated X proteins (Bax) in the heart tissues and the caspase family (caspase-3, 6 and 9). Additionally, RJ revealed a significant reduction in the expression of cardiac glycogen synthase kinase-3 (Gsk-3) and Nf-κB proteins [98].

### 5.1. Antihypertensive Activity of Royal Jelly

The biggest risk factor for cardiovascular disease around the globe is hypertension, which is caused by a disturbance in the contractile or proliferative function of the vascular smooth muscle cells of blood vessels [99].

#### 5.1.1. Preclinical Studies

Previous research has demonstrated that peptides isolated from RJ at doses of 1 g/kg considerably reduced the high blood pressure of hypertensive rats after 10 weeks of administration. The evidential effect was explained by the down-expression of angiotensin-1-converting enzyme, the main regulator of blood pressure [100]. The protein content of RJ represents half of its dry weight, while a major RJ protein, namely MRJP1-9, represents 80% of its total protein content [101]. A recent in vitro study revealed that incubation of aortic vascular smooth muscle cell lines from mice with MRJP1 showed a significant reduction in the cellular α-smooth muscle actin protein, the marker responsible for hypertension [102]. A similar in vivo study conducted on experimentally induced hypertension in Wistar rats using angiotensin-converting enzyme revealed that administration of RJ at a dosage of 15 mg/kg every day for four weeks to hypertensive rats suppressed their increases in blood pressure [103]. Another preclinical study on rats and rabbits showed that RJ exhibited a hypotensive impact via increased nitric oxide production. The study also demonstrated vasodilation effects through the suppression of the cyclic guanosine monophosphate pathway that mediates the contraction and relaxation of vascular smooth muscle cells [104]. Other studies suggested that a muscarinic receptor agonist, like acetylcholine, might be one of the vasodilators in RJ. In fact, it has already been noted that RJ contains more than 900 µg/g of acetylcholine-like substances. Additionally, acetylcholine stimulates the release of endothelium-derived relaxing factors (EDRFs), including nitric oxide (NO), prostacyclin (PGI2), and endothelium-derived hyperpolarizing factor (EDHF), by the vascular endothelial cells. They are the primary mediators of the vasorelaxant effects that are endothelium dependent [104,105].

#### 5.1.2. Clinical Studies

**Figure 5:**
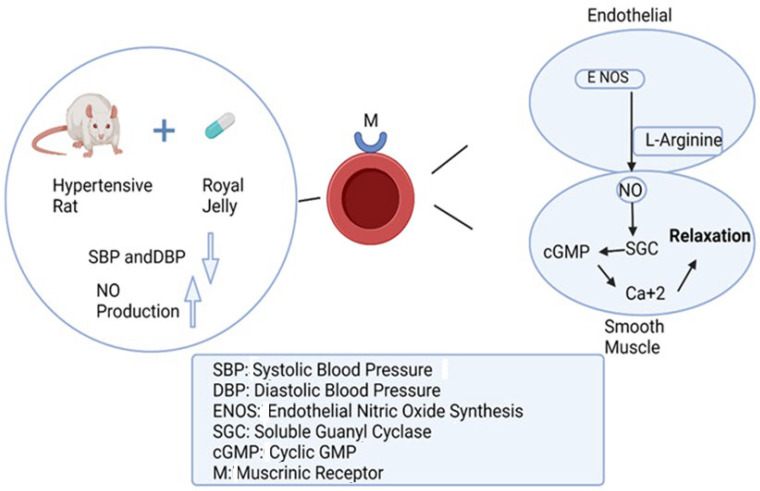
The mechanism of the antihypertensive activity of royal jelly.

At the clinical level, a randomized, placebo-controlled study demonstrated that a daily intake of RJ tablets (690 mg) for four weeks significantly improved the vascular endothelial activity of the participants’ blood vessels, suggesting that RJ may exert anti-atherogenic activity [106]. Another clinical study reported that the treatment of renal failure patients suffering from cardiovascular disease with a daily dosage of RJ (3600 mg) for a year considerably attenuated the progression of atherosclerosis in hemodialysis patients [15]. Figure 5 illustrates the mechanism of the antihypertensive activity of RJ.

### 5.2. Hypo-Cholesterolemic Activity of Royal Jelly

Cholesterol plays an important role in several physiological activities inside the body; nevertheless, increased levels in serum cause serious health problems, including cardiovascular disorders [107]. Previous studies reported that providing a diet containing 5% RJ to mice for seven days markedly reduced cholesterol levels in the blood through down-expression of squalene epoxidase, which is essential for the biosynthesis of cholesterol [108]. Animal experimentation proved that MRJP1 has anti-cholesterolemic potential through its interaction with bile acids that increase cholesterol catabolism in the liver and excretion of cholesterol in feces, compared to a β-sitosterol drug [109].

In a placebo-controlled trial, daily use of RJ capsules containing 350 mg RJ for three months was found to significantly alter low-density lipoprotein and total cholesterol levels [110]. The study’s findings led the authors to the conclusion that RJ consumption significantly increased the levels of dehydroepiandrosterone sulphate (DHEA-S). By significantly raising the concentration of DHEA-S over the course of three months, nine RJ capsules taken daily might significantly reduce the levels of LDL-c and total cholesterol (TC) in the serum. In addition, the investigations revealed that DHEA-S can affect the activity of glycerol 3-phosphate dehydrogenase and glucose 6-phosphate dehydrogenase, which in turn can stop the creation of NADPH and hence prevent the biosynthesis of fatty acids, phospholipids, and cholesterol. The hypo-cholesterolemic potential of RJ has been confirmed by a meta-analysis study that supported the notion that RJ reduces total cholesterol levels while also increasing high-density lipoproteins and accordingly regulating the lipid profile [16]. According to Balan et al. (2020), atherosclerosis and cardiovascular diseases related to post-menopausal symptoms in women were found to be inhibited with the regular intake of RJ [111].

## 6. Conclusions and Future Prospects

The beneficial impact of RJ on diabetes, gastrointestinal ailments, and cardiovascular disease is well-documented in the research articles reviewed. These findings, derived from both preclinical and clinical studies, highlight the potential of RJ as a promising intervention in the field of metabolic health. Furthermore, RJ may enhance the treatment of these disorders by mitigating the adverse effects linked with the drugs used for managing diabetes, gastrointestinal, and cardiovascular conditions. However, despite these promising findings, the specific mechanisms through which RJ exerts its therapeutic effects remain a subject of ongoing research. This underscores the need for further scientific investigations to validate the therapeutic efficacy of RJ and to fully understand its role in disease management. As we move forward, it is crucial to continue exploring the potential of RJ in treating metabolic disorders, with a focus on elucidating its mechanisms of action and potential applications in personalized medicine.

## Figures and Tables

**Table 1:** Bioactive compounds identified from royal jelly as antidiabetic agents.

Identified Compounds	Dosage	Biological Activity (In Vitro/In Vivo)	References
Hesperetin	40 mg/kg body weight (BW) for 45 days	Reduces high blood sugar and lipid levels by enhancing insulin secretion (in vivo).	[42,43]
Naringenin	(25, 50, 100 mg/kg) for 4 weeks	The therapy significantly enhanced the control of blood glucose levels and also contributed to the recovery of BWin diabetic rats, in contrast to those that received a vehicle treatment (in vivo).	[43,44]
	50 mg/kg for 4 weeks	Adequate to mitigate the alterations in the lenses caused by diabetes-related oxidative stress (in vivo).	[45]
	50 mg/kg/day for 5 days	Significant decrease in blood glucose and triglyceride levels in diabetic rats (in vivo).	[46]
	100 mg/kg BW /day for 4 weeks	Restored the serum insulin and C-peptide levels, replenished liver glycogen, and reduced glucose-6-phosphatase and glycogen phosphorylase activity in the liver. Additionally, it improved the serum lipid profile and strengthened the liver’s antioxidant defense system (in vivo).	[47]
Genistein	(20 and 40 mg/kg) for 8 weeks.	Improved glucose tolerance, blood glucose levels, insulin, glucagon, lipid profiles, and pro-inflammatory factors. It also improved liver function, reduced inflammation in the liver and colon, and positively altered gut microbiota composition (in vivo).	[43,48]
	25–200 mg/day	Improved hyperglycemia, glucose tolerance, and blood insulin levels, along with enhancing islet beta-cell proliferation, survival, and mass (in vivo).	[49]
	600 mg/kg for 4 weeks	Enhanced insulin sensitivity and increased expression of neurotrophic factors, such as nerve growth factor (NGF) and brain-derived neurotrophic factors (BDNF) (in vivo).	[50]
Formononetin	20 mg/kg for 28 days	Reduced serum glucose levels and increased serum insulin compared to the control group. It also decreased insulin resistance and reduced fasting glucose (C57BL/6 mice, in vivo).	[43,51]
	40 mg/kg/day for 16 weeks	Decreased insulin resistance and regulated hypoglycemia in male rats with diabetes (in vivo).	[52]
Coumestrol	50 µM	Improved hepatic insulin resistance in primary at hepatocyte (in vivo).	[43,53]
Chrysin	100 mg/kg	It resulted in a reduction of fasting blood glucose and insulin levels in db/db mice when compared to the control group (in vivo).	[43,54]
	80 mg/kg BW for 10 days	Anti-diabetic effects via increasing insulin levels, reducing oxidative stress, and regulating the inflammatory pathway (in vivo).	[55]
10-Hydroxy-2-decenoic acid	100 mg per kg BW/Daily for 4 weeks.	Decreased fasting blood glucose and increased insulin levels in diabetic mice. Enhanced activity of crucial antioxidants in the livers of diabetic mice, such as superoxide dismutase, catalase, and glutathione peroxidase (in vivo).	[39,56]
